# Supplementary material for: Association between hypomagnesemia and mortality among dialysis patients: a systematic review and meta-analysis
Source: PeerJ. 2022 Oct 11;10:e14203. doi: 10.7717/peerj.14203 (PMC9563282; doi:10.7717/peerj.14203)
Supplement: Supplemental Information 1 [file peerj-10-14203-s001.docx]

1. **Search equation via PubMed, EMBASE, and** [**Cochrane**](http://www.cochranelibrary.com/) **library**

**Appendix.**

Search strategies for the different databases ran on 2022.04.03

**PubMed Search Query**

"Renal Dialysis"[MeSH Terms] AND ("magnesium"[MeSH Terms] OR "magnesium"[All Fields] OR "magnesium s"[All Fields] OR "magnesiums"[All Fields] OR ("hypomagnesaemia"[All Fields] OR "hypomagnesemia"[All Fields]) OR ("hypomagnesaemia"[All Fields] OR "hypomagnesemia"[All Fields]) OR ("hypermagnesaemia"[All Fields] OR "hypermagnesemia"[All Fields]) OR ("hypermagnesaemia"[All Fields] OR "hypermagnesemia"[All Fields]))

**EMBASE**

1# (hemodialysis OR haemodialysis OR peritoneal) AND (hypomagnesemia OR magnesium OR hypermagnesemia)

#1 AND [embase]/lim NOT ([embase]/lim AND [medline]/lim)

**Cochrane Library**

#1 MeSH descriptor: [Dialysis] explode all trees

#2 dialysis

#3 Hemodialysis

#4 "Peritoneal dialysis"

#5 #1 OR #2 OR #3 OR #4

#6 hypomagnesemia

#7 magnesium

#8 hypermagnesemia

#9 #6 OR #7 OR #8

#10 #5 AND #9
