# Supplementary material for: Association between hypomagnesemia and mortality among dialysis patients: a systematic review and meta-analysis
Source: PeerJ. 2022 Oct 11;10:e14203. doi: 10.7717/peerj.14203 (PMC9563282; doi:10.7717/peerj.14203)
Supplement: Supplemental Information 2 [file peerj-10-14203-s002.docx]

**PROSPERO protocol registration**

International prospective register of systematic reviews

Systematic review

1. * Review title.

Give the title of the review in English

The impact of serum magnesium on mortality, and cardiovascular disease in dialysis patients: a systematic review and meta-analysis

2. Original language title.

For reviews in languages other than English, give the title in the original language.

This will be displayed with the English language title.

3. * Anticipated or actual start date.

Give the date the systematic review started or is expected to start.

20/05/2021

4. * Anticipated completion date.

Give the date by which the review is expected to be completed.

31/07/2021

5. * Stage of review at time of this submission.

Tick the boxes to show which review tasks have been started and which have been completed. Update this field each time any amendments are made to a published record.

Reviews that have started data extraction (at the time of initial submission) are not eligible for

inclusion in PROSPERO. If there is later evidence that incorrect status and/or completion date has been supplied, the published PROSPERO record will be marked as retracted.

This field uses answers to initial screening questions. It cannot be edited until after registration.

The review has not yet started: No

Review stage Started Completed

Preliminary searches, Yes

Piloting of the study selection process, Yes

Formal screening of search results against eligibility criteria, Yes

Data extraction, No

Risk of bias (quality) assessment, No

Data analysis, Yes

Provide any other relevant information about the stage of the review here.

6. * Named contact.

The named contact is the guarantor for the accuracy of the information in the register record. This may be any member of the review team.

Chi Ya Huang

Email salutation (e.g. "Dr Smith" or "Joanne") for correspondence: Dr Huang

7. * Named contact e-mail, sheila21928@gmail.com

8. Named contact address

No. 901, Zhonghua Rd., Yongkang Dist., Tainan City 710402 , Taiwan (R.O.C.)

9. Named contact phone number.

086-978177386

10. * Organisational affiliation of the review.

Chi Mei Medical center

Organisation web address: http://www.chimei.org.tw/

11. * Review team members and their organisational affiliations.

Dr Chi-Ya Huang. Chi Mei Medical center

Dr Chi-Chen Yang. Chi Mei Medical Center

Dr Jui-Yi Chen. Chi Mei Medical Center

Dr Yun-Ting Huang. Chi Mei Medical Center

12. * Funding sources/sponsors.

Details of the individuals, organizations, groups, companies or other legal entities who have funded or sponsored the review.

nil

Grant number(s)

State the funder, grant or award number and the date of award, nil

13. * Conflicts of interest.

None

14. Collaborators.

Give the name and affiliation of any individuals or organisations who are working on the review but who are not listed as review team members. NOTE: email and country must be completed for each person, unless you are amending a published record.

15. * Review question.

State the review question(s) clearly and precisely. It may be appropriate to break very broad questions down into a series of related more specific questions. Questions may be framed or refined using PI(E)COS or similar where relevant.

P: Patients on dialysis

I: Hypomagnesemia or normomagnesemia

C: Hypermagnesemia

O: All cause mortality or CV mortality

The impact of serum magnesium on mortality, and cardiovascular disease in dialysis patients: a systematic review and meta-analysis

16. * Searches.

State the sources that will be searched (e.g. Medline). Give the search dates, and any restrictions (e.g.language or publication date). Do NOT enter the full search strategy (it may be provided as a link or attachment below.)

PubMed, Cochrane, Embase

17. URL to search strategy.

Upload a file with your search strategy, or an example of a search strategy for a specific database, (including the keywords) in pdf or word format. In doing so you are consenting to the file being made publicly accessible. Or provide a URL or link to the strategy. Do NOT provide links to your search results.

Search: ((((((("Renal Dialysis"[Mesh]) OR (Dialysis)) OR (hemodialysis)) OR (peritoneal dialysis)) OR(haemodialysis)) AND (magnesium)) OR (hypomagnesemia)) OR (hypermagnesemia) (("Renal Dialysis"[MeSH Terms] OR ("dialysance"[All Fields] OR "dialysances"[All Fields] OR "dialysation"[All Fields] OR "dialysator"[All Fields] OR "dialysators"[All Fields] OR "dialyse"[All Fields] OR "dialysed"[All Fields] OR "dialyser"[All Fields] OR "dialysers"[All Fields] OR "dialysing"[All Fields] OR "dialysis solutions"[Pharmacological Action] OR "dialysis solutions"[MeSH Terms] OR ("dialysis"[All Fields] AND "solutions"[All Fields]) OR "dialysis solutions"[All Fields] OR "dialysate"[All Fields] OR "dialysates"[All Fields] OR "dialyzate"[All Fields] OR "dialyzates"[All Fields] OR "dialysis"[MeSH Terms] OR "dialysis"[All Fields] OR "dialyses"[All Fields] OR "dialyzability"[All Fields] OR "dialyzable"[All Fields] OR "dialyzation"[All Fields] OR

"dialyze"[All Fields] OR "dialyzed"[All Fields] OR "dialyzer"[All Fields] OR "dialyzer s"[All Fields] OR "dialyzers"[All Fields] OR "dialyzing"[All Fields] OR "Renal Dialysis"[MeSH Terms] OR ("renal"[All Fields] AND "dialysis"[All Fields]) OR "Renal Dialysis"[All Fields]) OR ("haemodialysis"[All Fields] OR "Renal Dialysis"[MeSH Terms] OR ("renal"[All Fields] AND "dialysis"[All Fields]) OR "Renal Dialysis"[All Fields] OR "hemodialysis"[All Fields]) OR ("peritoneal dialysis"[MeSH Terms] OR ("peritoneal"[All Fields] AND "dialysis"[All Fields]) OR "peritoneal dialysis"[All Fields]) OR ("haemodialysis"[All Fields] OR "Renal

Dialysis"[MeSH Terms] OR ("renal"[All Fields] AND "dialysis"[All Fields]) OR "Renal Dialysis"[All Fields] OR "hemodialysis"[All Fields])) AND ("magnesium"[MeSH Terms] OR "magnesium"[All Fields] OR "magnesium" [All Fields] OR "magnesiums"[All Fields])) OR ("hypomagnesaemia"[All Fields] OR "hypomagnesemia"[All Fields]) OR ("hypermagnesaemia"[All Fields] OR "hypermagnesemia"[All Fields])

18. * Condition or domain being studied.

Give a short description of the disease, condition or healthcare domain being studied in your systematic review.

Mortality in dialysis patients

19. * Participants/population.

Specify the participants or populations being studied in the review. The preferred format includes details of both inclusion and exclusion criteria.

Dialysis patients, including hemodialysis and peritoneal dialysis

20. * Intervention(s), exposure(s).

Hypomagnesemia and normomagnesemia

21. * Comparator(s)/control.

Hypermagnesemia

22. * Types of study to be included.

Retrospective or prospective cohort studies

23. Context.

Give summary details of the setting or other relevant characteristics, which help define the inclusion or exclusion criteria.

Search RCTs containing data of serum magnesium and the relationship of all cause mortality and/or CV mortality from the group of patients undergoing renal replacement therapy

24. * Main outcome(s).

All cause mortality

Measures of effect

Use Hazard Ratio and Confidence interval for data analysis

25. * Additional outcome(s).

List the pre-specified additional outcomes of the review, with a similar level of detail to that required for main outcomes. Where there are no additional outcomes please state ‘None’ or ‘Not applicable’ as appropriate to the review

CV mortality(death due to IHD, arrhythmia, cardiac sudden death, congestive heart failure, other heart disease or cerebrovascular events)

Measures of effect

Use Hazard Ratio and Confidence interval for data analysis

26. * Data extraction (selection and coding).

Cohort studies containing data of serum magnesium and the relationship of all-cause mortality and/or CV mortality from the group of patients undergoing renal replacement therapy Need data of HR or KM curve of all cause or CV mortality

27. * Risk of bias (quality) assessment.

Use Newcastle-Ottawa scale

28. * Strategy for data synthesis.

Analyze collection data of HR of all cause mortality and CV mortality, and the relationship of serum magnesium

29. * Analysis of subgroups or subsets.

Subgroups of sex(proportion of men), study design, renal replacement therapy modality

30. * Type and method of review.

Type of review

Cost effectiveness, No

Diagnostic, No

Epidemiologic, No

Individual patient data (IPD) meta-analysis, No

Intervention, No

Living systematic review, No

Meta-analysis, Yes

Methodology, No

Narrative synthesis, No

Network meta-analysis, No

Pre-clinical, No

Prevention, No

Prognostic, Yes

Prospective meta-analysis (PMA), No

Review of reviews, No

Service delivery, No

Synthesis of qualitative studies, No

Systematic review, Yes

Health area of the review

Alcohol/substance misuse/abuse, No

Blood and immune system, No

Cancer, No

Cardiovascular, Yes

Care of the elderly, No

Child health, No

Complementary therapies, No

COVID-19, No

Crime and justice, No

Dental, No

Digestive system, No

Ear, nose and throat, No

Education, No

Endocrine and metabolic disorders, No

Eye disorders, No

General interest, No

Genetics, No

Health inequalities/health equity, No

Infections and infestations, No

International prospective register of systematic reviews, No

Mental health and behavioural conditions, No

Musculoskeletal, No

Neurological, No

Nursing, No

Obstetrics and gynaecology, No

Oral health, No

Palliative care, No

Perioperative care, No

Physiotherapy, No

Pregnancy and childbirth, No

Public health (including social determinants of health), No

Rehabilitation, No

Respiratory disorders, No

Service delivery, No

Skin disorders, No

Social care, No

Surgery, No

Tropical Medicine, No

Urological, No

Wounds, injuries and accidents, No

Violence and abuse, No

31. Language, English

32. * Country, Taiwan

33. Other registration details.

34. Reference and/or URL for published protocol.

No I do not make this file publicly available until the review is complete

35. Dissemination plans.

Do you intend to publish the review on completion? No

36. Keywords.

serum magnesium, hypomagnesemia, hypermagnesemia, renal replacement therapy, hemodialysis, dialysis, mortality, death

37. Details of any existing review of the same topic by the same authors.

38. * Current review status.

Review_Ongoing

39. Any additional information, nil

40. Details of final report/publication(s) or preprints if available, nil
