# Supplementary material for: Association between hypomagnesemia and mortality among dialysis patients: a systematic review and meta-analysis
Source: PeerJ. 2022 Oct 11;10:e14203. doi: 10.7717/peerj.14203 (PMC9563282; doi:10.7717/peerj.14203)
Supplement: Supplemental Information 3 [file peerj-10-14203-s003.docx]

**Quality assessment of the included studies**

**Supplement Table 1. Newcastle-Ottawa Scale Quality Assessment of included studies**

|  | **Selection** | | | | | **Comparability** | | **Exposure** | | | | |  |
| --- | --- | --- | --- | --- | --- | --- | --- | --- | --- | --- | --- | --- | --- |
| First author (Year) | **Representativeness of the exposed cohort** | | **Selection of the non-exposed cohort** | **Ascertainment of exposure** | **Demonstration that outcome of interest was not present at start of study** | **Comparability of cohorts on the basis of the design or analysis** | | **Assessment of outcome** | **Was follow-up long enough for outcomes to occur** | | | **Adequacy of follow up of cohorts** | **Total** |
| Ishimura (2007) | * | * | | * | * | | ** | * | | * | * | | 9 |
| Markaki (2012) | * | * | | * | * | |  | * | | * | * | | 7 |
| Broek (2013) | * |  | | * | * | | * | * | | * | * | | 7 |
| Matias (2014) | * | * | | * | * | | * | * | | * | * | | 8 |
| Fein (2015) | * | * | | * | * | | ** | * | | * | * | | 9 |
| Lacson (2015) | * |  | | * | * | | ** | * | | * | * | | 8 |
| de Roij van  Zuijdewijn (2015) | * |  | | * | * | | ** | * | | * | * | | 9 |
| Garagarza (2015) | * |  | | * | * | | ** | * | |  |  | | 6 |
| Kurita (2015) | * |  | | * | * | | ** | * | |  | * | | 7 |
| Li (2016) | * | * | | * | * | | ** | * | | * | * | | 9 |
| Yang (2016) | * | * | | * | * | | ** | * | | * | * | | 9 |
| Cai (2016) | * | * | | * | * | | ** | * | | * | * | | 9 |
| Ago (2016) | * | * | | * | * | | * | * | |  | * | | 7 |
| Selim (2017) | * | * | | * | * | | * | * | | * | * | | 8 |
| Ye (2018) | * | * | | * | * | | * | * | | * | * | | 8 |
| Wu (2019) | * | * | | * | * | | ** | * | | * | * | | 9 |
| Tamuru (2019) | * | * | | * | * | | * | * | | * | * | | 8 |
| Mizuirui-2 (2019) | * | * | | * | * | | * | * | | * | * | | 8 |
| Shimohata (2019) | * |  | | * | * | | * | * | | * | * | | 7 |
| Mizuirui (2020) | * | * | | * | * | | * | * | | * | * | | 8 |
| Lu (2020) | * | * | | * | * | | * | * | |  | * | | 7 |
