# Supplementary figures and images for: Association between hypomagnesemia and mortality among dialysis patients: a systematic review and meta-analysis
Source: PeerJ. 2022 Oct 11;10:e14203. doi: 10.7717/peerj.14203 (PMC9563282; doi:10.7717/peerj.14203)

**The Grade results**

**
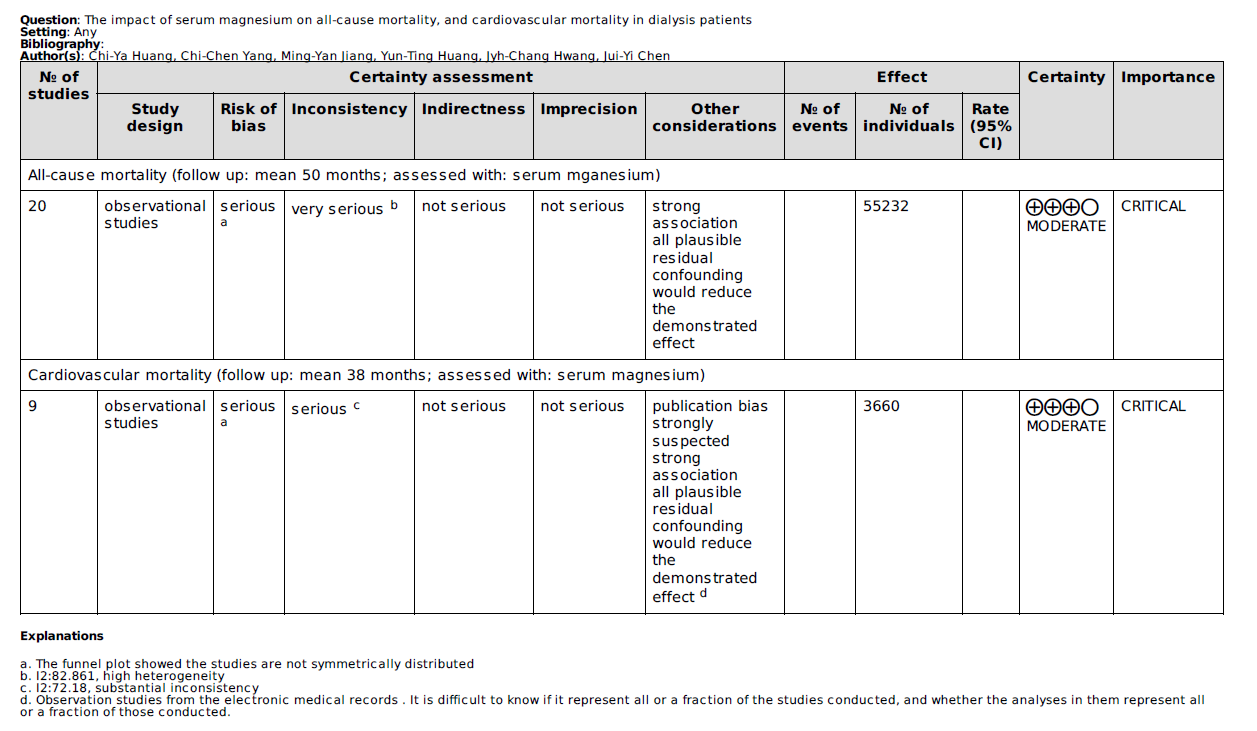
**

Supplement: Supplemental Information 5 [file peerj-10-14203-s005.docx]

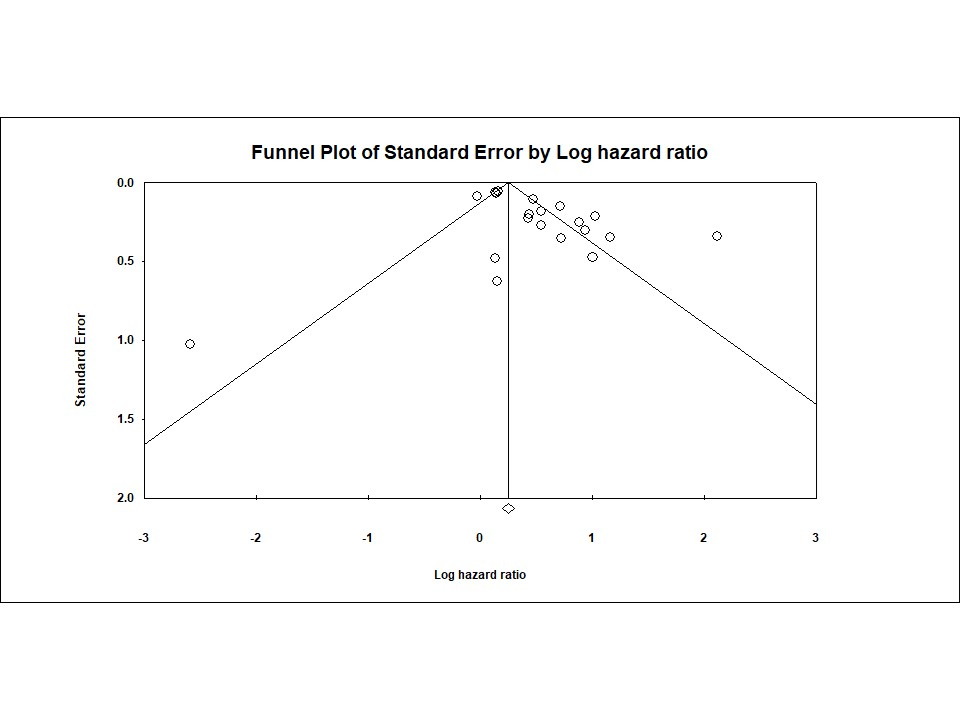

Supplement: Supplemental Information 7 [file peerj-10-14203-s007.png]

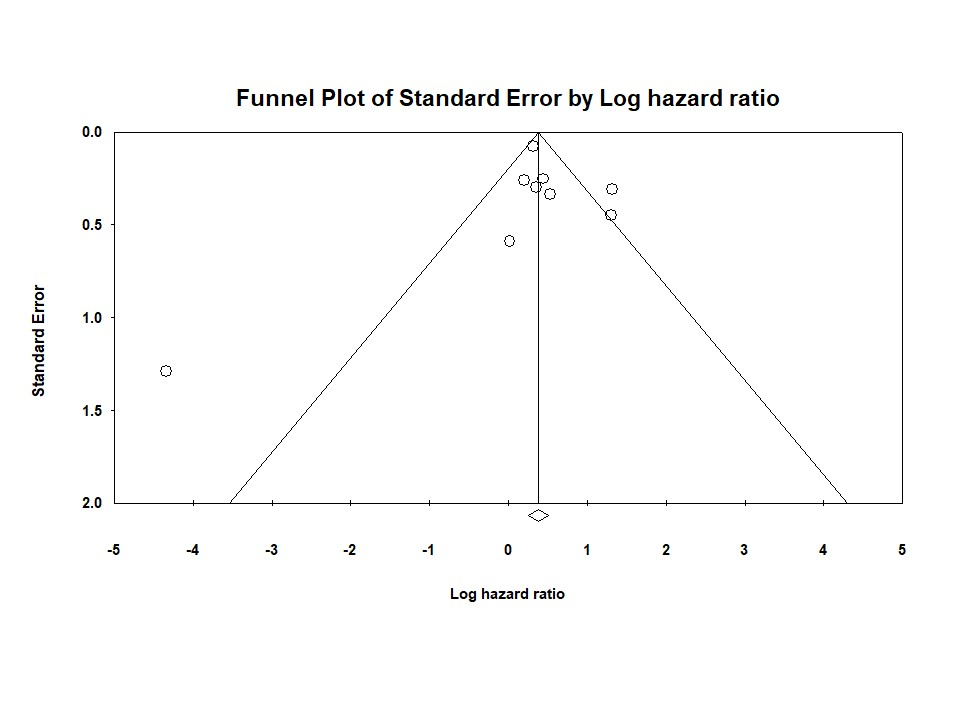

Supplement: Supplemental Information 8 [file peerj-10-14203-s008.png]

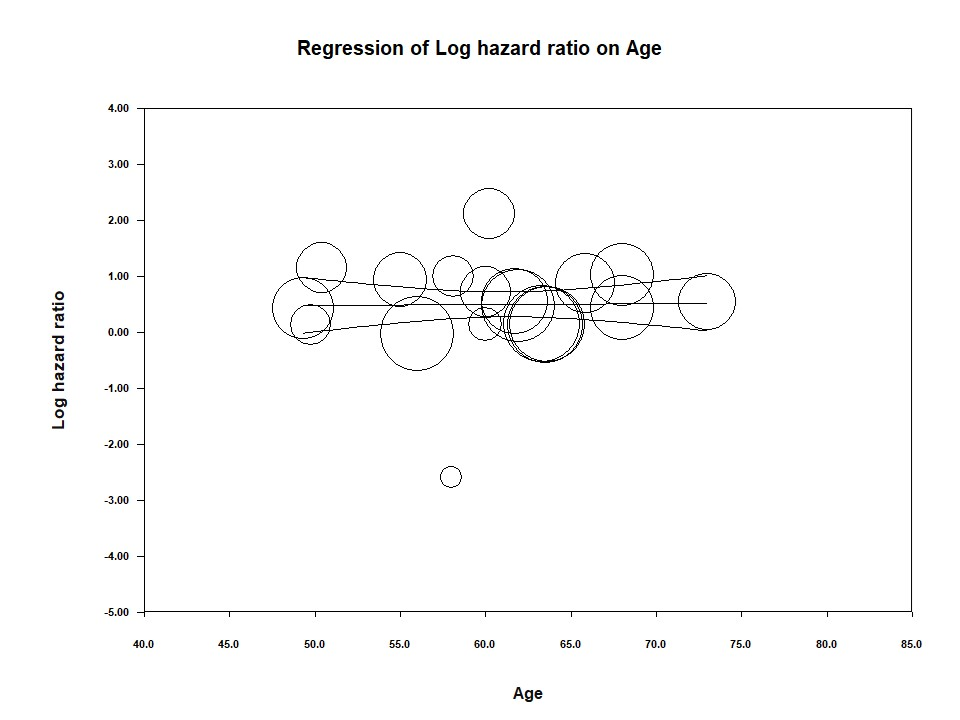

Supplement: Supplemental Information 9 [file peerj-10-14203-s009.png]

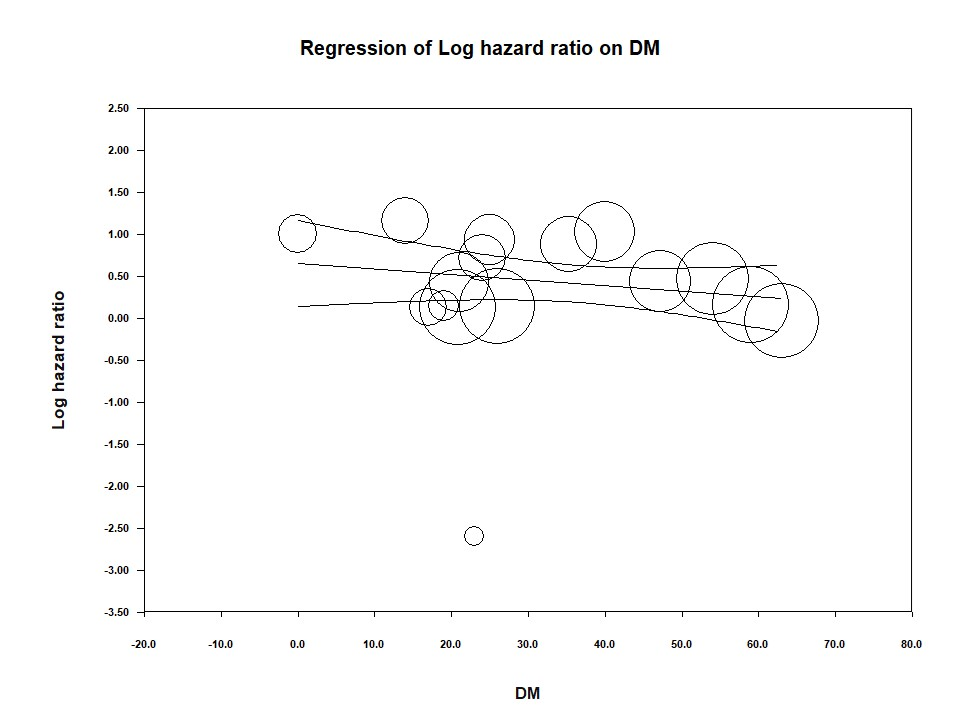

Supplement: Supplemental Information 10 [file peerj-10-14203-s010.png]

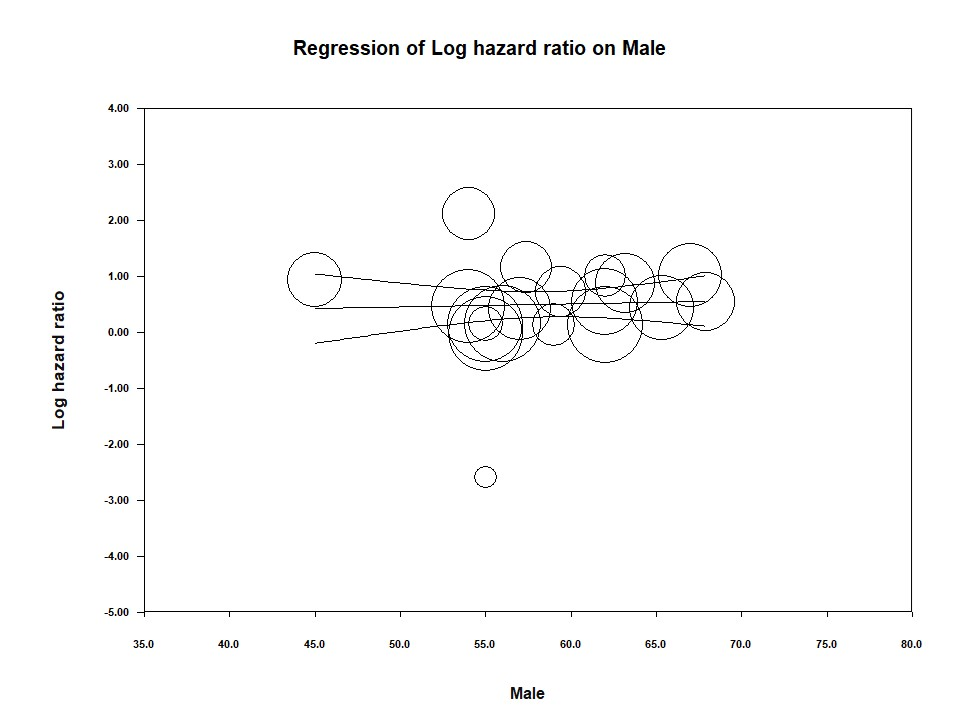

Supplement: Supplemental Information 11 [file peerj-10-14203-s011.png]

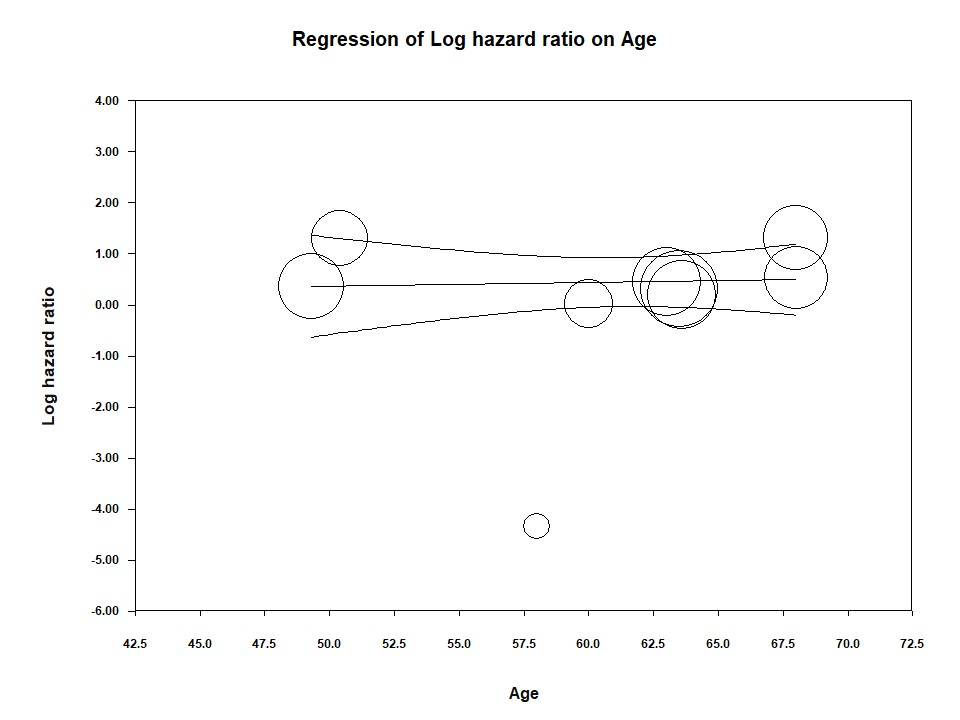

Supplement: Supplemental Information 12 [file peerj-10-14203-s012.png]

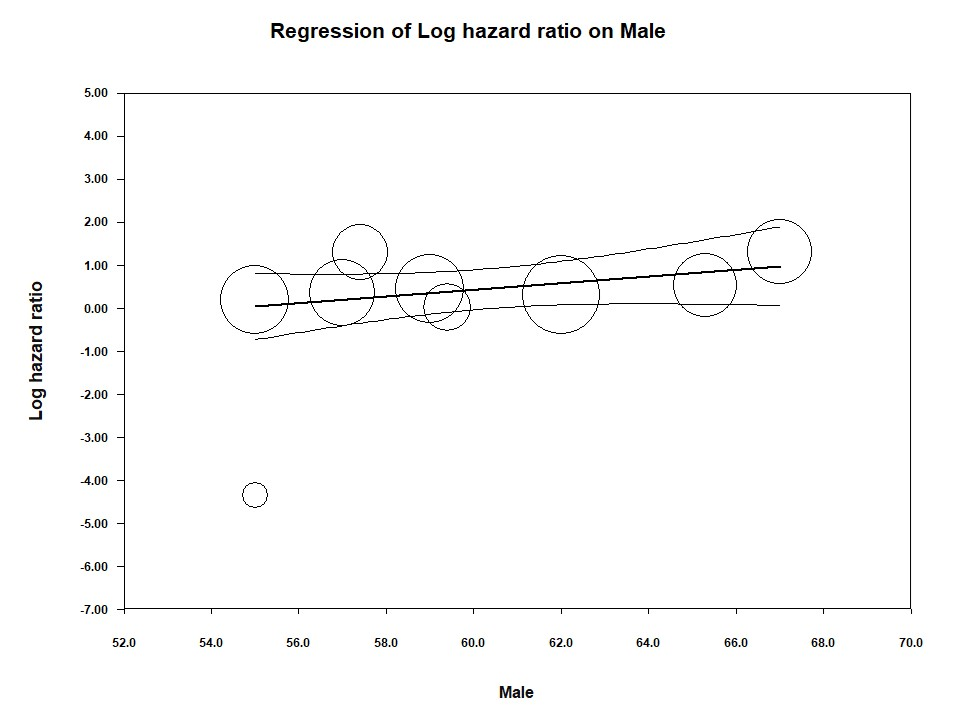

Supplement: Supplemental Information 13 [file peerj-10-14203-s013.png]

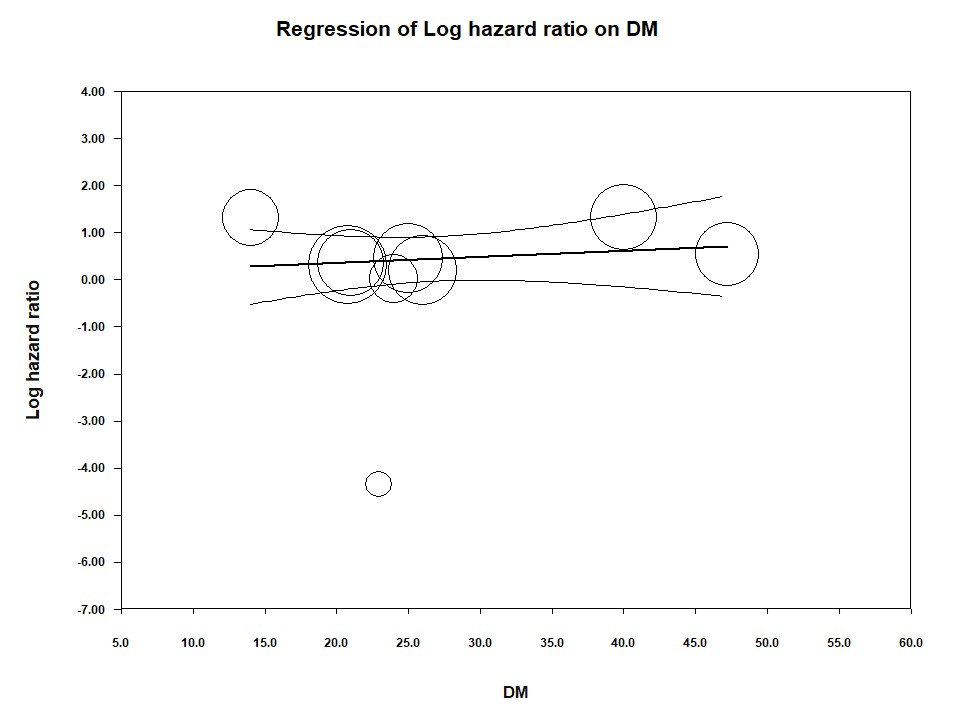

Supplement: Supplemental Information 14 [file peerj-10-14203-s014.png]
