# Supplementary material for: Association between hypomagnesemia and mortality among dialysis patients: a systematic review and meta-analysis
Source: PeerJ. 2022 Oct 11;10:e14203. doi: 10.7717/peerj.14203 (PMC9563282; doi:10.7717/peerj.14203)
Supplement: Supplemental Information 6 [file peerj-10-14203-s006.docx]

**Table S4 Meta-regression analysis**

| **Moderators** | **Coefficient** | **SE** | **Z value** | **P value** | **95% CI** |
| --- | --- | --- | --- | --- | --- |
| **All-cause mortality** | | | | | |
| The quantitative measures of serum levels of Mg | 0.2103 | 0.2218 | 0.95 | 0.3432 | -0.2245-0.6451 |
| Age | 0.0018 | 0.0153 | 0.12 | 0.90 | -0.0282-0.0319 |
| prevalence of diabetes | -0.0066 | 0.0052 | -1.27 | 0.205 | -0.0167-0.0036 |
| male proportion | 0.0061 | 0.0172 | 0.35 | 0.72 | -0.0277-0.0399 |
| **CV mortality** | | | | | |
| The quantitative measures of serum levels of Mg | 0.4996 | 0.5498 | 0.91 | 0.3636 | -0.5781-1.5772 |
| Age | 0.0091 | 0.0308 | 0.29 | 0.7685 | -0.0513-0.0694 |
| prevalence of diabetes | 0.0148 | 0.0199 | 0.75 | 0.4548 | -0.0241-0.0538 |
| male proportion | 0.0799 | 0.0473 | 1.69 | 0.0907 | -0.0127-0.1726 |

Abbreviation: CI, confidence interval; Mg, Magnesium; SE, standard error
