# Supplementary material for: Association between hypomagnesemia and mortality among dialysis patients: a systematic review and meta-analysis
Source: PeerJ. 2022 Oct 11;10:e14203. doi: 10.7717/peerj.14203 (PMC9563282; doi:10.7717/peerj.14203)
Supplement: Supplemental Information 15 [file peerj-10-14203-s015.docx]

**Systematic Review and Meta-Analysis Rationale for “Association between hypomagnesemia and mortality among dialysis patients: a systematic review and meta-analysis”**

1. **The rationale for conducting the systematic review / meta-analysis**

The impact of serum magnesium concentration on the outcomes for the different population is still unclear. For example, hypermagnesium for hospitalized patients is a strong predictor of poor outcomes compared with hypomagnesium individuals. (Cheungpasitporn et al. 2015) However, higher magnesium level is beneficial for the survival rate among the patients with chronic kidney disease(CKD). (Kanbay et al. 2012) For the dialysis patients, we are still not sure about the effect of different serum magnesium level on the all-cause/cardiovascular(CV) mortality. Therefore, we want to investigate the association between the serum magnesium level and mortality for dialysis patients.

1. **The contribution that it makes to knowledge in light of previously published related reports, including other meta-analyses and systematic reviews.**

Xiong et al. made a systematic review and meta-analysis about serum magnesium, mortality, and CV disease in chronic kidney disease and end-stage renal disease(ESRD) patients.(Xiong et al. 2019) However, we consider the effect of serum magnesium on the CKD and dialysis patients is different. The ultrafiltration rate of the magnesium, fluctuation of magnesium concentration before/after dialysis are the factors related to the outcomes. Therefore, we investigated the association between the serum magnesium and all-cause/CV mortality for only dialysis patients.

On the other hand, Liu et al.(Liu & Wang 2021) also had a meta-analysis about the association between the serum magnesium level and all-cause/CV outcomes for CKD and ESRD patients. The impact of serum magnesium on peritoneal dialysis (PD) and hemodialysis patients was not discussed separately. In our study, we aimed to evaluate the all-cause mortality, CV mortality who had hypomagnesemia versus non-hypomagnesemia among dialysis patients. We investigated the impact of serum magnesium on peritoneal dialysis and hemodialysis separately. In addition, we also analyze the diverse cut-off value of serum magnesium for the included studies. Our study suggested lower magnesium concentration had a significant risk of all-cause mortality and CV mortality compared with non-hypomagnesemia within dialysis participants. Hypomagnesemia had more impact on HD patients than PD patients for clinical outcomes.

**Reference**

Cheungpasitporn W, Thongprayoon C, and Qian Q. 2015. Dysmagnesemia in Hospitalized Patients: Prevalence and Prognostic Importance. *Mayo Clin Proc* 90:1001-1010. 10.1016/j.mayocp.2015.04.023

Kanbay M, Yilmaz MI, Apetrii M, Saglam M, Yaman H, Unal HU, Gok M, Caglar K, Oguz Y, Yenicesu M, Cetinkaya H, Eyileten T, Acikel C, Vural A, and Covic A. 2012. Relationship between serum magnesium levels and cardiovascular events in chronic kidney disease patients. *Am J Nephrol* 36:228-237. 10.1159/000341868

Liu H, and Wang R. 2021. Associations between the serum magnesium and all-cause or cardiovascular mortality in chronic kidney disease and end-stage renal disease patients: A meta-analysis. *Medicine (Baltimore)* 100:e27486. 10.1097/md.0000000000027486

Xiong J, He T, Wang M, Nie L, Zhang Y, Wang Y, Huang Y, Feng B, Zhang J, and Zhao J. 2019. Serum magnesium, mortality, and cardiovascular disease in chronic kidney disease and end-stage renal disease patients: a systematic review and meta-analysis. *J Nephrol* 32:791-802. 10.1007/s40620-019-00601-6
